# In Plain Sight: The Need for a Dedicated Cardiovascular Infectious Disease Subspecialty

**DOI:** 10.1016/j.jacadv.2023.100748

**Published:** 2023-11-30

**Authors:** Sami El-Dalati, Molly L. Paras, Luke Strnad, Andrew W. Harris, John C. Gurley, Michael Sekela, Varidhi Nauriyal, James L. Januzzi, Doreen DeFaria Yeh, Evin Yucel

**Affiliations:** a Division of Infectious Diseases, Department of Internal Medicine, University of Kentucky College of Medicine, Lexington, Kentucky, USA; b Division of Infectious Diseases, Department of Internal Medicine, Massachusetts General Hospital, Harvard Medical School, Boston, Massachusetts, USA; c Division of Infectious Diseases, Department of Internal Medicine, Oregon Health and Science University, Portland, Oregon, USA; d Division of Cardiology, Department of Internal Medicine, Michigan Medicine, University of Michigan Health, Ann Arbor, Michigan, USA; e Division of Cardiology, Department of Internal Medicine, University of Kentucky College of Medicine, Lexington, Kentucky, USA; f Department of Cardiac Surgery, University of Kentucky College of Medicine, Lexington, Kentucky, USA; g Division of Infectious Diseases, Department of Internal Medicine, University of Pittsburgh Medical Center, Pittsburgh, Pennsylvania, USA; h Division of Cardiology, Department of Internal Medicine, Massachusetts General Hospital, Harvard Medical School, Boston, Massachusetts, USA; i Heart Failure and Biomarker Trials, Baim Institute for Clinical Research, Boston, Massachusetts, USA; j Division of Cardiology, Department of Internal Medicine, Massachusetts General Hospital, Harvard Medical School, Boston, Massachusetts, USA

**Keywords:** cardiac implantable electronic device, cardiovascular infections, endocarditis, left ventricular assist device, medical education, multidisciplinary teams, substance use disorder

Cardiovascular infections (CVIs) are associated with significant in-hospital and long-term morbidity and mortality. Despite antimicrobial advancements, along with the advent of cardiopulmonary bypass and valve surgery, in-hospital mortality from CVIs still ranges between 10 and 20%.^1^ Compounding this, the incidence of CVIs is growing: the incidence of native valve endocarditis increased by 21% between 2002 and 2016, peaking at ∼39,000 cases per year.^1^ Additionally, the proportion of cases associated with injection drug use increased from 11.9% to 20.8% during this period.^1^ Incidence and possibly complexity of CVIs is also expected to increase: new surgical and transcatheter treatments to address valvular heart disease, septal defects, arrhythmias, and cardiomyopathies have increased the number of patients at risk for CVI.

In 2020, there were ∼182,000 valve replacements in the United States, while annually over 200,000 cardiac implantable electronic devices (CIEDs) are placed.^2^^,^^3^ The incidence of prosthetic valve endocarditis (PVE) is 0.3 to 1.2% per patient per year and the incidence of CIED infection is approximately 1% at 12 months postimplantation.^3^^,^^4^ While early device extraction for CIED infection is associated with decreased mortality, the majority of patients with indications do not undergo extraction.^5^ Between 2010 and 2019, 25,551 left ventricular assist devices (LVADs) were implanted worldwide for cardiomyopathies, with 3,198 placed in 2019.^6^ One in 6 patients will develop an LVAD infection in the first year after placement and infection is responsible for 15% of all LVAD-related deaths.^6^

Patients with CVIs are generally medically complex, and existing literature demonstrates that patients with endocarditis are best cared for by multidisciplinary teams comprised of providers from a number of specialties.^7^^,^^8^ Currently, no advanced training pathways exist for providers interested in caring for patients with CVIs despite a high volume of affected patients and the nuanced care these individuals require. We suggest there is a need for physicians with expertise in infectious diseases (IDs), cardiology, and addiction medicine who can provide expert care and coordinate, communicate, and lead impactfully the medical and surgical subspecialists integral to the successful treatment of CVIs.

## Endocarditis

Despite advances in cardiovascular imaging modalities, microbiologic diagnostics, evidence supporting oral antibiotic treatment and the benefits of surgical intervention, in-hospital mortality of infectious endocarditis remains high.^1^ A specialty dedicated to treatment of CVIs creates an ideal opportunity to appropriately update the management of patients with infectious endocarditis, while decreasing length of stay, health care costs and mortality.

Deeper knowledge of the various imaging modalities that have emerged as useful in PVE is a good example of where specific training would be of value. Cardiac positron emission tomography (PET) and cardiac computerized tomography (CT) have high sensitivity for detection of PVE and its complications, which may be missed by echocardiography and require surgical intervention. CVI providers would have the epidemiologic knowledge and receive training in advanced cardiovascular imaging, allowing them to appropriately select patients for cardiac PET and/or CT as part of an evaluation for endocarditis. Additional cardiology training with regards to management of acute valvular regurgitation in the septic patient, surgical indications, and even management of valvular disease in pregnancy would all benefit a potential CVI physician.

A CVI training program would also provide a platform for up-to-date knowledge of treatment options for endocarditis. Both the 2015 American Heart Association and European Society of Cardiology Endocarditis Guidelines advocate for the use of intravenous (IV) antibiotic therapy for 4 to 6 weeks. Historically, there have been concerns that oral antibiotics would not sufficiently sterilize valvular vegetation. However, recent high-quality evidence suggests partial oral antibiotic treatment may be an appropriate alternative to long-term IV antibiotics in certain patients.^9^ Iversen et al demonstrated, in a 400-patient randomized controlled trial, that de-escalating patients with left-sided endocarditis to oral antibiotics after at least 10 days of IV treatment was non-inferior to completing a full course of IV therapy. Despite this, widespread utilization of oral antibiotic treatment for endocarditis has yet to occur. While IV therapy can be completed outpatient, this necessitates placement of a peripherally inserted central catheter which carries risk of infection and thromboembolism. Outpatient parenteral antibiotic therapy (OPAT) requires patients have stable housing or disposition to a medical facility capable of administering IV medications. Arranging reliable transportation and home health nursing for postdischarge follow-up can be challenging. Consequently, patients who inject drugs or have other structural vulnerabilities are often deemed ineligible to receive OPAT and are required to choose between remaining inpatient for 4 to 6 weeks to receive IV antibiotics or receiving no treatment. Additionally, retrospective literature has demonstrated that select patients undergoing valve surgery for endocarditis can complete 2-week postoperative courses of antibiotics with low rates of relapsed infection.^10^ A standardized, coordinated approach to transitioning patients to oral antibiotics or truncated postoperative courses led by dedicated CVI specialists could allow for significant reductions in length of stay and OPAT costs while increasing access to equitable treatment.

Perhaps the most impactful role for a CVI specialist involves creating and leading institutional and regional multidisciplinary teams which can decrease the in-hospital mortality for patients with endocarditis.^7^^,^^8^ Such collaborations are significant undertakings that require coordination between multiple medical and surgical subspecialties. The CVI-trained physician can serve as the highly informed liaison between providers, create health system best practice guidelines, and implement new initiatives.

CVI specialists could also receive additional training in substance use disorders (SUDs). There have been calls for a combined IDs addiction medicine training pathway to integrate the treatment of infections in patients who inject drugs. Expanding this model to a CVI provider would allow patients to follow-up with 1 physician for a host of their clinical needs. A patient with endocarditis could see 1 CVI specialist for treatment of endocarditis, chronic hepatitis B, C, or HIV, medication for opioid use disorder and follow-up of their valvulopathy.

## CIED and LVAD Infections

Despite the availability of organizational treatment recommendations for CIED infections, new evidence suggests that there are low rates of guideline adherence which are associated with increased patient mortality.^5^ While the reasons for this are unclear, there are inherent challenges in the health care system that may contribute. A much larger number of physicians are capable of implanting CIEDs than are trained to perform complex extractions, which carry risk of significant complications. Many patients who receive a transvenous CIED may have been candidates for a less invasive device such as a leadless pacemaker or subcutaneous defibrillator which are associated with lower rates of infection. Patients may present to emergency departments or primary care offices with limited experience in diagnosis and management of CIED infections. Additionally, there are gaps in the literature regarding when further evaluation for CIED infection is indicated and whether device removal is recommended. The limited sensitivity of diagnostic imaging modalities such as transesophageal echocardiography and PET does not allow for consistent identification of patients who would most benefit from CIED extraction. Consequently, physicians are often left weighing the risks of device extraction against those of relapsed or progressive infection. Currently, many ID and cardiology providers may not receive sufficient education to adequately assess both sets of risks.

Similarly, device infections affect a number of patients with LVADs and are a significant cause of LVAD-related mortality.^6^ Unlike CIED extractions, LVAD exchanges have high rates of associated mortality which lead to a large number of patients with LVAD infections on long-term antimicrobial suppression. These strategies, while often necessary, unfortunately contribute to antibiotic resistance and breakthrough infection which has high rates of morbidity and mortality, management challenges that could be best addressed by dedicated CVI specialists.

A CVI physician would also be trained to identify patients requiring further evaluation for CIED/LVAD infection and/or endocarditis, assess which patients would benefit from additional procedures and appropriately select patients for implantations of less-invasive devices. CVI providers would interface with physicians from referring hospitals to select patients who require transfer to tertiary care centers for CIED extraction, percutaneous mechanical aspiration of vegetations, or valve surgery and craft management strategies from afar for patients who are at prohibitively high risk for procedures and require long-term medical therapy.^8^

## Training pathway

Given the need for a group of subspecialists with advanced expertise in both ID and cardiology, a formal training pathway would create a standardized education process that annually produces cohorts of these providers. The ideal CVI fellowship would be available to ID providers and cardiologists. Currently these specialists, respectively, complete 2- or 3-year American College of Graduate Medical Education approved fellowship. One- to 2-year subspecialized training programs already exist for transplant ID and HIV care as well as 1- to 2-year cardiology fellowships in advanced heart failure, structural and interventional cardiology and electrophysiology. Following this model, a CVI fellowship could be completed in 1 year and could be housed in either the divisions of infectious disease or cardiology depending on the resources available at each institution. The optimal training program would reside in a tertiary care referral center with ID, addiction medicine, cardiology, and cardiac surgery expertise that can perform valve replacements, CIED, and LVAD explants as well as heart transplantation (Table 1). This would allow the CVI fellow to potentially spend time with experts in transplant ID and cardiac transplantation, further complementing their education. The center should also have a multidisciplinary endocarditis team that connects the aforementioned providers. Depending on their initial training background, fellows could spend varying amounts of time rotating with ID providers and working with cardiovascular imaging specialists. If an institution does not have a dedicated CVI consult service, these cases could be preferentially seen by the fellow and staffed with the on-call ID attending to maximize trainee exposure. All fellows would spend time with addiction providers to gain familiarity with SUDs and trauma informed care. Additionally, trainees would have the opportunity to observe CIED implantations and extractions, cardiac surgeries and percutaneous tricuspid valve aspirations. Longitudinal follow-up could be conducted in individual specialty clinics and/or a multidisciplinary clinic staffed by the aforementioned specialties. Optimally, the trainee would have a once weekly continuity clinic in which they could see patients and provide SUD treatment.

**Table 1:** Proposed Training Program for a Cardiovascular Infectious Diseases Fellowship Including Specific Rotations and Their Duration, Minimum Requirements and ACGME Core Competencies

	Goal of Training			
	Minimum Requirements	Selected Skills	Duration of Rotation	ACGME Core Competency Addressed
Infectious diseases	*Knowledge of appropriate antimicrobial testing and therapeutics for cardiovascular infection*			Patient Care (PC) Medical Knowledge (MK) Interpersonal and Communication Skills (IPC) Professionalism (P) Practice-Based Learning and Improvement (PBLI) Systems-Based Practice (SBP)
	• Longitudinal clinic >40 wk/y	• Management of antimicrobial therapy, including outpatient parenteral antibiotic therapy (OPAT)	4 mo inpatient rotation (ideally with cardiac ID consult service)	
	• Didactics	• Recognize complications of therapy		
		• Recognize signs/symptoms of recurrent infection		
		• Screen for comorbid infectious diseases (HIV, Hep C)		
Multimodality cardiovascular imaging	*Recognize appropriate use of cardiac imaging modalities*			MK IPC SBP
	*Basic competency in viewing TTE and TEE*			
	*Conversant with field of cardiac PET imaging*			
	*Understand principles, indications, and limitation of cardiac CT*			
	• Interpret 100 TTE/TEEs	• Integrate basic TTE and TEE images in clinical practice	• 1-mo rotation in echo lab (TTE/TEE)	
	• Interpret 30 cardiac CTs	• Integrate cardiac PET studies in patient care	• 1-mo rotation in advanced cardiac imaging (PET/CT)	
	• Interpret 15 cardiac PETs	• Integrate cardiac CT into the diagnosis and management of endocarditis		
	• Didactics			
CIED infection	*Recognize indications for CIED placement, including the role of leadless devices*			MK
	*Recognize signs/symptoms of CIED infection*			
	*Identify indications for device removal*			
	• Observe 3 device removals	• Integrate knowledge of CIEDs and CIED infections into clinical care	• 1-mo rotation in device clinic/observing device placement and extractions	
	• Didactics			
LVAD infection	*Recognize indications for LVAD placement and cardiac transplantation*			PC MK ICS P PBLI SBP
	*Differentiate between types of LVAD infection (driveline, pump)*			
	• Care for 10 patients with LVAD infections	• Integrate knowledge of LVAD infections into clinical care	• 1-mo rotation in transplant infectious diseases (optional)	
	• Care for 5 cardiac transplant patients (optional)		• 1-mo rotation (optional) advanced heart failure	
Addiction medicine	*Recognize signs/symptoms of and screen for substance use disorder*			PC MK ICS P PBLI SBP
	*Basic understanding of treatment with medications for opioid use disorder*			
	*Understand principles of destigmatizing language*			
	• Observe/manage 15 buprenorphine inductions	• Perform straightforward buprenorphine inductions	1-mo inpatient rotation	
	• Longitudinal clinic >40 wk/y^a^	• Recognize indications to refer for complex addiction treatment		
	• Didactics	• Basic knowledge of existing local SUD treatment programs		
Multidisciplinary team care	*Recognize role of multidisciplinary endocarditis teams in patient care*			PC ICS P PBLI SBP
	*Understand core principles of development of a multidisciplinary team*			
	*Independently lead multidisciplinary team meetings*			
	• Lead 5 multidisciplinary meetings	• Participate in/Lead a multidisciplinary team meeting	Weekly multidisciplinary endocarditis team meetings	
	• Longitudinal multidisciplinary clinic >10/y			
	• Observe 3 cardiac surgeries			
	• Observe 3 percutaneous tricuspid valve debulkings			
Scholarly activity	*Participate in scholarly activity leading to conference poster/abstract submission, quality improvement initiatives, presentation, teaching activity, or publication*			ICS PBLI SBP

The text in italics emphasizes the general goals of training for that particular discipline. The non-italicized text highlights the specific requirements for each rotation.

ACGME = American College of Graduate Medical Education; CIED = cardiac implantable electronic device; CT = computerized tomography; ICS = interpersonal and communications skills; ID = infectious disease; LVAD = left ventricular assist device; PET = positron emission tomography; SUD = substance use disorder; TEE = transesophageal echocardiography; TTE = transthoracic echocardiography.

a Addiction treatment would ideally occur concurrently in the infectious diseases clinic. In the proposed pathway, the trainee would be in clinic approximately 1 ½ day per week.

## Conclusions

While ideally patients with CVIs would be managed effectively by interfacing teams of providers independently trained in ID, addiction medicine, cardiology, and cardiac surgery, most institutions in the United States do not have established multidisciplinary teams to provide optimal CVI management. Existing data suggest the current standard of care contributes to significant patient morbidity and mortality, health care inequities and increases in length of stay and spending. High rates of antimicrobial resistance in conjunction with the large numbers of individuals with injection drug use, prosthetic valves, CIEDs, and LVADs create progressively complex care scenarios that require subspecialized expertise to manage successfully. A dedicated CVI instructional program accessible to physicians with multiple training backgrounds offers a pathway to provide multidisciplinary leaders and streamline treatment for a population greatly in need of improved care.

## Funding support and author disclosures

The authors have reported that they have no relationships relevant to the contents of this paper to disclose.
